# Psychological Distress in Adults With Myocardial Infarction: Implications for Health Care Utilization and Expenditure

**DOI:** 10.1016/j.jacadv.2024.101540

**Published:** 2025-01-09

**Authors:** Harshith Thyagaturu, Ola Abdelhadi, Hafiz Muhammad Waqar Younas, Mohamed Abugrin, Vikram Padala, Lalitsiri Atti, Tala Altarawneh, Vijaykumar Sekar, Karthik Gonuguntla, Sudarshan Balla, Martha Gulati

**Affiliations:** a Department of Cardiology, West Virginia University Heart and Vascular Institute, Morgantown, West Virginia, USA; b Office of Research Patient Care Services, Stanford Health Care, Palo Alta, California, USA; c Department of Internal Medicine, Weiss Memorial Hospital, Chicago, Illinois, USA; d Department of Internal Medicine, Bassett Medical Center, Cooperstown, New York, USA; e Lewis Katz School of Medicine at Temple University, Philadelphia, Pennsylvania, USA; f Department of Internal Medicine, Sparrow Hospital- Michigan State University, Lansing, Michigan, USA; g Department of Internal Medicine, Marshall University, Huntington, West Virginia, USA; h Department of Endocrinology, Lehigh Valley Health Network, Allentown, Pennsylvania, USA; i Department of Cardiology, Barbra Streisand Women’s Heart Center, Cedars Sinai- Smidt Heart Institute, Los Angeles, California, USA; j The Baim Institute for Research, Boston, Massachusetts, USA

**Keywords:** health care utilization, medical expenditure, mental health, MEPS, myocardial infarction, psychological distress

## Abstract

**Background:**

Myocardial infarction (MI) poses a major financial burden on the U.S. health care system, but its impact on medical expenses and health care utilization when coupled with psychological distress remains unknown.

**Objectives:**

The study aims to investigate the association between psychological distress and healthcare utilization and medical expenditures in adults with a history of MI.

**Methods:**

We analyzed the 2017-2021 Medical Expenditure Panel Survey to identify 44,716 adults with a history of MI. Psychological distress was measured using the Kessler (K6) questionnaire, with a score of ≥13 indicating clinically significant distress. Differences in medical expenditures and health care utilization between patients with MI with and without psychological distress were assessed using weighted generalized linear and negative binomial regression models. Expenditures, medical visits, and prescribed medications are reported as means and 95% CI.

**Results:**

Among 9,773,458 weighted adults, 970,049 experienced psychological distress. Adults with MI and psychological distress were younger, more likely to be female (51.1% vs 37.5%; *P* < 0.001), less educated (11.1 vs 12.5 years; *P* < 0.001), lower income, and were more likely to have public insurance, compared to those without psychological distress. Adults with psychological distress and a history of MI had higher average medical expenses ($31,577 vs $15,830; *P* < 0.001) and greater health care utilization including office visits (8.3 vs 5.7; *P* = 0.01), inpatient visits (0.6 vs 0.3; *P* < 0.001), emergency room visits (0.7 vs 0.3; *P* < 0.001), and prescribed medications including refills (42.3 vs 28; *P* < 0.001).

**Conclusions:**

Psychological distress is correlated with increased medical expenditures and health care utilization in patients with MI. This research highlights the need for interventions addressing psychological needs in patients with MI.

Cardiovascular diseases, particularly coronary artery disease (CAD), are among the leading causes of mortality in the United States, incurring an estimated annual cost of $407.3 billion.^1^ Psychological distress, in the form of generalized anxiety, depression, and panic disorder, is common among patients with myocardial infarction (MI).^2^ Pathophysiological mechanisms for depression in patients with MI include dysregulation of the hypothalamic-pituitary-adrenal axis, coagulation system dysfunction, inflammation, as well as genetic factors.^3^ Up to 41% of MI population have concomitant depression, which has a strong impact on the progression of coronary heart disease and yet remains relatively under-recognized.^4^^,^^5^ In addition, depression in those with MI significantly increases the risk of mortality and cardiac events.^6^^,^^7^ A connection between psychological distress and the recurrence of myocardial ischemia in CAD population has also been described, with suggested mechanisms involving endothelial dysfunction and platelet reactivity.^8^, ^9^, ^10^ Psychological distress in CAD can cause a decline in their quality of life and a rise in unhealthy behaviors like medication nonadherence, resulting in increased adverse events.^11^ Our study aims to explore the relationship between psychological distress and medical expenditures, as well as health care utilization, in individuals with a history of MI. In addition, we aim to identify demographic and socioeconomic factors associated with psychological distress in those with a history of MI.

## Methods

### Data source

We used the publicly available Medical Expenditure Panel Survey-Household Component (MEPS-HC) data from January 2017 to December 2021. The MEPS is a large-scale survey of a nationally representative sample of noninstitutionalized families and individuals, their medical providers, and employers across the United States.^12^ The MEPS is sponsored by the Agency for Healthcare Research and Quality, and the sample was taken from a panel of individuals who participated in the prior year’s National Health Interview Survey (NHIS) conducted by the National Center for Health Statistics. During the household interviews, MEPS collects data on each person in the household regarding demographics, health conditions, use of medical services, charges and source of payment, access to care, health insurance coverage, income, and employment. Every year, a new panel of sample households is selected. There are 5 interview rounds across 2 years, and data are collected through in-person, virtual interviews (during the COVID-19 pandemic) and self-administered questionnaires. This study uses deidentified publicly available data, and hence the Institutional Review Board approval was not obtained. However, the MEPS project has been reviewed and approved by the Westat Institutional Review Board, established under a multiproject assurance (MPA M-1531) granted by the Office for Protection from Research Risks. Approval for the project is renewed annually.

### Study population

**Figure 1:**
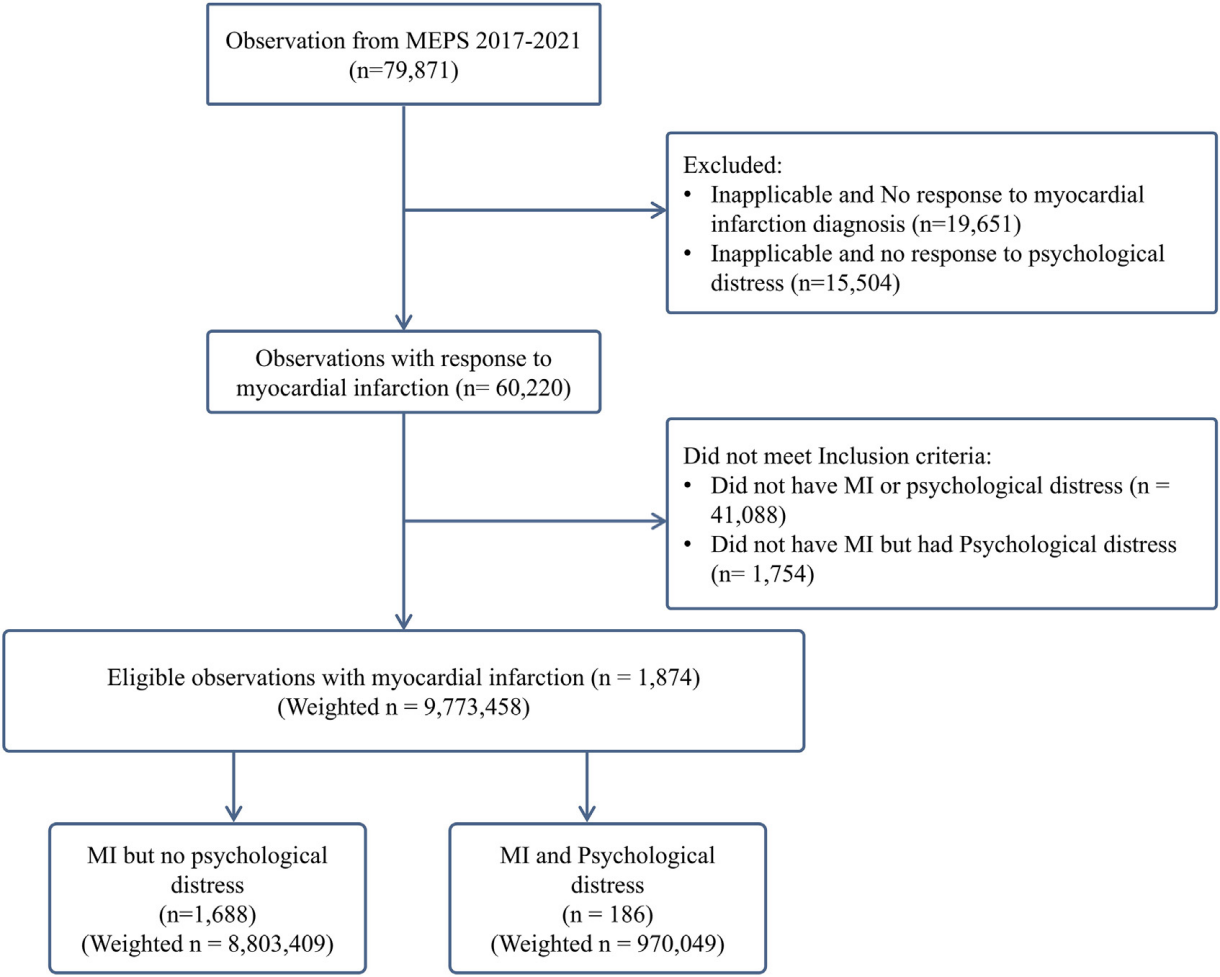
**Study Flow Chart** Flow chart depicting the selection process for study participants from the 2017 to 2021 medical expenditure panel survey data. Starting with 79,871 observations, exclusions were made for inapplicable or missing responses related to myocardial infarction and psychological distress. The final sample included 1,874 participants with myocardial infarction, divided into groups with and without psychological distress. Weighted sample sizes are shown for context. MEPS = Medical Expenditure Panel Survey; MI = myocardial infarction.

The reporting of the study follows the Strengthening the Reporting of Observational Studies in Epidemiology (STROBE) reporting guideline (Supplemental Appendix). We identified adult (>18 years) participants who were diagnosed with MI using the priority condition survey question or International Classification of Diseases, Tenth Revision, Clinical Modification (ICD-10-CM) code, “I21.” The complete list of ICD-10-CM codes used in this study is detailed in Supplemental Table 1. Psychological distress was assessed using the Kessler (K6) questionnaire, a widely validated screening tool for identifying nonspecific psychological distress.^13^^,^^14^ In the survey, the participants were asked 6 questions assessing the frequency of symptoms such as feeling nervous, hopeless, restless, or worthless over the past 30 days. Responses are scored from 0 to 4, yielding a maximum score of 24. A score of 13 or higher indicates clinically significant psychological distress.^15^ The specific questionnaire and answer options are shown in the Supplemental Appendix. The K6 questionnaire has demonstrated excellent internal consistency with Cronbach’s alpha values typically between 0.85 to 0.89 for general population,^13^ and 0.78 to 0.85 in those with chronic conditions including those with cardiovascular conditions,^16^ supporting its applicability in evaluating psychological distress in patients with MI,^17^^,^^18^ how they had been feeling during the past 30 days. Adults were excluded without history of MI, inapplicable or missing responses regarding MI (n = 19,651), and psychological distress (n = 15,504), resulting in 1,874 eligible participants. Figure 1 details the selection of our patient population.

### Outcomes

The primary outcome was to assess the difference in medical expenditure in those with prior MI based on psychological distress status. Medical expenditures were calculated as the U.S. dollar amount spent within different clinical settings like ambulatory visits, inpatient admissions, emergency visits, prescription medications, and other expenses averaged per year. Secondary outcomes assessed were differences in health care utilization, which were measured as the number of office-based provider visits, emergency room (ER) visits, inpatient nights, hospital discharges, and prescribed medications including refills per year. MEPS relies on a series of interviews with participants to gather detailed data on health care usage, expenditures, sources of payment, and insurance coverage. To improve the accuracy of this self-reported data, MEPS also includes a Medical Provider Component where data from health care providers, hospitals, and pharmacies are collected to verify and supplement participants' reports.

### Covariates

Using MEPS-HC components, we collected the demographics of each participant: age, sex, race, region of residence, ethnicity, family income, insurance coverage, marital status, and education status. Demographic MEPS-HC components are collected through self-reported data from survey participants during structured interviews. An elevated body mass index was defined as >25 kg/m^2^, and adverse health behaviors included smoking (currently smoking) and not exercising regularly (not meeting moderate exercise 5 times a week). We merged the demographics file with the medical conditions file before identifying those with a history of MI and other comorbidities. Using the Federal Poverty Level (FPL) as the reference entity, 5 categories of family income were identified: poor/negative (≤100% of FPL), near poor (100% to 125% of FPL), low income (125% to 200% of FPL), middle income (200% to 400% of FPL), and high income (>400% of FPL). The FPL represents an income threshold set by the Department of Health and Human Services that varies by household size and is adjusted annually to account for inflation and changes in the cost of living. 100% of the FPL means a household earns exactly at the poverty line. 200% of the FPL means a household earns twice the poverty-level income. This percentage is crucial for understanding financial vulnerability and determining eligibility for income-based assistance programs. Chronic conditions like coronary heart disease, hypertension, diabetes mellitus, angina, stroke, high cholesterol, cancer, and asthma were preidentified as priority conditions within the MEPS data set. Other important comorbidities like anxiety, depression, obstructive sleep apnea along with other sleep disorders were identified using ICD-10-CM codes provided within the medical conditions file. In addition, the comorbidity burden was assessed using the Charlson comorbidity index scoring system.

### Statistical analysis

All statistical analyses were performed using Stata, version 18.0 SE-Standard Edition (StataCorp). Stata facilitates MEPS design complexities that include stratification, clustering, and weighting to produce national estimates. After downloading the necessary MEPS-HC files, we restructured the medical conditions file to create person-level data and merged it with a full-year consolidated file to create the annual file. To assess baseline characteristics differences between groups, we used the Student’s t-test for continuous variables and the chi-square test for nominal/categorical variables. The dependent variable followed a gamma distribution, and various link functions (identity, log, and gamma) were tested to determine the best-fitting model based on the Akaike Information Criterion. The gamma regression model relates the mean expenditure to a set of predictors through a log-transformed linear relationship. Additionally, we confirmed that events were independent, intervals did not overlap, and the probability of more than 1 event occurring in a short period was approximately 0. We employed weighted gamma regression with a log link to model health care expenditures, which are continuous, positively skewed, and non-negative. This model assumes that the mean expenditure μ (mu) is related to a set of predictors via a log link function: log(μ) = β₀ + β₁x₁ + … + βkxk; where μ represents the mean of the gamma distribution, and β₀, β₁, … βk are the regression coefficients associated with the predictor variables x₁ … xk. For people with x₁ (MI vs no-MI), the expected value of the response variable (μ[expenses]) is multiplied by exp (β₁). To get expenses in $: exp (β₀+ β₁) − exp (β₀). To estimate office-based visits and prescription medications including refills, weighted multivariable regression models with negative binomial distributions and log links were used. To assess inpatient nights and ER visits, given that a large percentage of participants did not use these services, zero-inflation negative binomial models were used. All models were adjusted for the following: age at survey, sex, race, education, insurance, income, marital status, exercise, body mass index, smoking status, CAD, hypertension, diabetes mellitus, heart failure, chronic obstructive pulmonary disease, high cholesterol, cancer, and asthma. With an R-square of 86.5%, the gamma regression model explains a substantial portion of the variance in medical expenditures, effectively capturing variations across the sample while adjusting for relevant predictors. To evaluate differences of psychological distress in patients with MI with demographics, socioeconomic, behavioral factors, and comorbidities, a fully saturated multivariable logistic regression model (adjusted for all covariates) was used and adjusted ORs (aORs) with CIs were obtained. We applied all survey weights and used survey analysis tools that include strata, clusters, Primary Sampling Units in addition to the weights. Missing values were excluded, and percentages are based on the number of nonmissing values. However, the total proportion of missing values were <3%.

## Results

### Demographics and baseline comorbidities

A total of 1,874 (weighted n = 9,773,458) adults with MI were identified from January 2017 to December 2021, of which, 186 (weighted = 970,049) had concomitant psychological distress and 1,688 (weighted = 8,803,409) did not (Figure 1). The overall weighted prevalence of psychological distress was higher in persons with MI compared to persons without MI (9.3% vs 3.6%; *P* < 0.001). Furthermore, the average K6 score for psychological distress in the MI population was 4.25, whereas it was 2.74 in the population without MI. We found a decreasing trend in the prevalence of psychological distress among adults with MI from 2017 to 2020 (11.7% in 2017 vs 3.7% in 2020) and noticed a steep increase in 2021 (10.5%) (Supplemental Table 2). Adults with MI and psychological distress were more likely female (51.1% vs 37.5%; *P* < 0.001), with a mean age of 63.1 ± 12.5 years and a higher proportion of Hispanic ethnicity (19.3% vs 11.5%; *P* = 0.002) compared to adults with MI and no psychological distress. Furthermore, adults with MI and psychological distress more commonly had less than high school education (41.9% vs 27.3%; *P* < 0.001), divorced (30.6% vs 21.6%; *P* = 0.02), ≤100% of FPL (38.1% vs 16.9%; *P* < 0.001), and public insurance (73.6% vs 51.5%; *P* < 0.001) compared to adults with MI and no psychological distress. Comorbidities in adults with MI that had statistically significant differences with psychological distress included hypertension, diabetes mellitus, hyperlipidemia, chronic obstructive pulmonary disease (emphysema or chronic bronchitis), asthma, anxiety, and depression. Table 1 shows detailed baseline demographics and comorbidities.

**Table 1:** Baseline Characteristics of the Study Population: Medical Expenditure Panel Survey 2017-2021

	MI With Psychological Distress (n = 186)	MI Without Psychological Distress (n = 1,688)	*P* Value
Age, y	63.13 ± 12.5	67.56 ± 12.4	<0.001
Age group, y			0.001
18-39	7 (3.76%)	43 (2.55%)	
40-64	90 (48.39%)	582 (34.48%)	
65-85	80 (43.01%)	909 (53.85%)	
≥85	9 (4.84%)	154 (9.12%)	
Female	95 (51.08%)	633 (37.50%)	<0.001
Race			0.002
White	110 (59.14%)	1,156 (68.48%)	
Black	23 (12.37%)	241 (14.28%)	
Hispanic	36 (19.35%)	194 (11.49%)	
Asian and other	17 (9.14%)	97 (5.74%)	
Region			0.24
Northeast	23 (12.37%)	268 (15.88%)	
Midwest	41 (22.04%)	438 (25.95%)	
South	79 (42.47%)	661 (39.16%)	
West	43 (23.12%)	321 (19.02%)	
Years of education	11.10 ± 4.32	12.48 ± 3.42	<0.001
Highest degree			<0.001
Less than high school	78 (41.94%)	461 (27.31%)	
High school graduate	77 (41.40%)	735 (43.54%)	
College or more	31 (16.67%)	492 (29.15%)	
Marital status			0.024
Married	74 (39.78%)	836 (49.53%)	
Widowed	35 (18.82%)	319 (18.90%)	
Divorced	57 (30.65%)	365 (21.62%)	
Never married	20 (10.75%)	168 (9.95%)	
Health insurance			
Private	41 (22.04%)	775 (45.91%)	<0.001
Public	137 (73.66%)	870 (51.54%)	
Uninsured	8 (4.30%)	43 (2.55%)	
Income			<0.001
Poor	71 (38.17%)	286 (16.94%)	
Near poor	24 (12.90%)	135 (8.00%)	
Low income	39 (20.97%)	292 (17.30%)	
Middle income	30 (16.13%)	483 (28.61%)	
High income	22 (11.83%)	492 (29.15%)	
Smoking	42 (22.58%)	284 (16.84%)	0.05
Exercise regularly	47 (27.17%)	705 (42.68%)	<0.001
Unable to get care when needed	22 (17.74%)	145 (14.96%)	0.66
BMI >25 kg/m^2^	172 (92.47%)	1,520 (90.05%)	0.28
Comorbidities			
Coronary heart disease	117 (62.90%)	1,077 (63.99%)	0.09
Angina	51 (27.42%)	394 (23.37%)	0.22
Hypertension	165 (88.71%)	1,351 (80.04%)	0.004
Diabetes mellitus	86 (46.24%)	616 (36.49%)	0.009
COPD (emphysema or chronic bronchitis)	42 (22.58%)	222 (13.15%)	<0.001
High cholesterol	153 (82.70%)	1,263 (74.82%)	0.02
Cancer	54 (29.03%)	424 (25.13%)	0.25
Asthma	60 (32.26%)	295 (17.48%)	<0.001
Diagnosed with depression	80 (43.01%)	192 (1.37%)	0.001
Diagnosed with anxiety	64 (34.41%)	220 (13.03%)	<0.001
Heart failure	17 (9.14%)	127 (7.52%)	0.43

Values are mean ± SD or n (%).

BMI = body mass index; COPD = chronic obstructive pulmonary disease.

### Impact of psychological distress on medical expenditure and health care utilization

Our findings revealed that those with MI and psychological distress had higher annual per-person total medical expenditure compared to those with MI without psychological distress ($31,577 vs $15,831; *P* < 0.001) indicating approximately $15,746.68 associated with the presence of psychological distress. Similarly, we found a higher average annual per-person expenditures for inpatient ($23,527 vs $11,224; *P* = 0.008), ER-based ($1,526.6 vs $808.3; *P* = 0.001), and medication (5,797.5 vs 2921.9; *P* < 0.001) in those with MI and psychological distress compared to persons with MI without psychological distress (Table 2). Those persons with MI and psychological distress had a significantly higher number of yearly office-based visits (8.3 vs 5.7; *P* = 0.01), inpatient hospitalizations (0.6 vs 0.3; *P* < 0.001), ER visits (0.76 vs 0.34; *P* < 0.001), and number of medications + refills (42.3 vs 27.9; *P* < 0.001), compared to persons with MI without psychological distress (Table 3). Additionally, we conducted a comparison of average expenses and health care utilization for persons without a history of MI with and without psychological distress as illustrated in the accompanying Tables 2 and 3.

**Table 2:** Medical Expenses for Myocardial Infarction and Non-Myocardial Infarction Adults With and Without Psychological Distress

Expenses	MI With Psychological Distress	MI Without Psychological Distress	*P* Value	No-MI With Psychological Distress	No-MI Without Psychological Distress	*P* Value
Total medical	$31,577.5 (23,386.6-42,637.1)	$15,830.8 (8,798.1-28,485.4)	<0.001	$6,149.25 (5,443.40-6,946.64)	$2,576.20 (2,048.55-3,239.77)	<0.001
Office-based visits	$7,061.3 (3,761.9-13,254.5)	$4,705.3 (1,956.4-11,316.2)	0.21	$969.65 (858.05-1,095.76)	$515.60 (362.78-732.80)	<0.001
Inpatient visits	$23,527.6 (13,620.1-40,642.1)	$11,224.2 (3,395.6-37,101.8)	0.008	$3,634.89 (2,709.82-4,875.75)	$1,103.21 (619.40-1,964.93)	<0.001
ER visits	$1,526.9 (1,054.5-2,211.1)	$808.5 (295.1-2,215.9)	0.001	$860.85 (718.49-1,031.41)	$368.34 (231.77-585.38)	<0.001
Medications (total)	$5,753.37 (3,946.8-8,386.8)	$2,918.04 (1,517.1-5,612.4)	<0.001	$1,276.03 (1,039.29-1,566.71)	$459.70 (298.61-707.71)	<0.001
Medications (out-of-pocket)	$273.52 (187.8-398.4)	$185.17 (93.6-368.1)	0.04	$117.34 (98.49-139.81)	$54.28 (32.04-91.94)	<0.001

Values are mean (95% CI). Expenditures are expressed in US$.

ER = emergency room; MI = myocardial infarction.

**Table 3:** Health Care Utilization and Number of Medication With Refills for Myocardial Infarction and Nonmyocardial Infarction Adults With and Without Psychological Distress

	MI With Psychological Distress	MI Without Psychological Distress	*P* Value	No-MI With Psychological Distress	No-MI Without Psychological Distress	*P* Value
No. of office-based visits	8.27 (6.2-11.1)	5.74 (3.4-9.6)	0.01	3.1 (2.7-3.4)	1.4 (1.2-1.7)	<0.001
No. of inpatient visits	0.59 (0.4-0.8)	0.28 (0.1-0.6)	<0.001	0.09 (0.08-0.1)	0.03 (0.02-0.04)	<0.001
No. of ER visits	0.76 (0.6-0.9)	0.34 (0.2-0.6)	<0.001	0.23 (0.20-0.26)	0.09 (0.07-0.11)	<0.001
No. of medication refills	42.32 (36.9-48.4)	27.98 (19.8-39.3)	<0.001	6.03 (5.5-6.5)	2.1 (1.8-2.5)	<0.001

Values are mean (95% CI).

Abbreviations as in Table 2.

### Predictors of psychological distress in persons with MI

**Figure 2:**
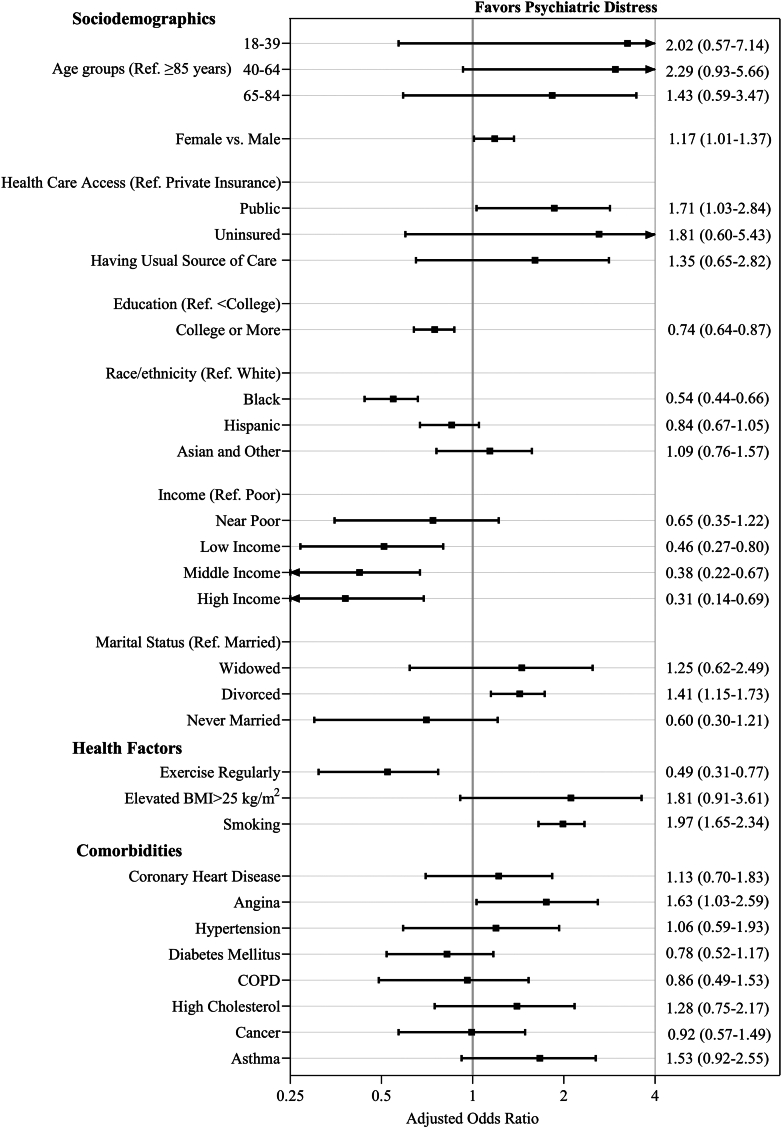
**Sociodemographics, Behavioral, and Comorbidity Predictors Associated With Psychological Distress in Participants With Myocardial Infarction** Forest plot showing adjusted odds ratios and confidence intervals for predictors of psychological distress in myocardial infarction patients. Factors like female sex, smoking, and angina are linked to higher odds of distress, while regular exercise, higher income, and college education are associated with lower odds, illustrating the influence of demographic and health factors. Note: usual source of care is the particular medical professional, doctor's office, clinic, health center, or other place where a person would usually go if sick or in need of advice about his or her health. BMI = body mass index; COPD = chronic obstructive pulmonary disease.

After adjusting for confounding variables and accounting for survey weights, psychological distress in persons with MI was more likely to be associated with public insurance vs private (aOR: 1.71 [95% CI: 1.03-2.84]; *P* = 0.04), females vs males (aOR: 1.17 [95% CI: 1.01-1.37]; *P* = 0.04), divorced vs married (aOR: 1.41 [95% CI: 1.15-1.73]; *P* = 0.001), participants who often smoked (aOR: 1.97 [95% CI: 1.65-2.34]; *P* < 0.001) and with reported history of angina (aOR: 1.63 [95% CI: 1.03-2.59]; *P* = 0.04) and asthma (aOR: 1.63 [95% CI: 1.10-2.41]; *P* = 0.02) (Figure 2, Supplemental Table 3). Psychological distress in persons with MI was less likely to be associated with Black persons compared to White persons (aOR: 0.54 [95% CI: 0.44-0.66]; *P* = 0.001), participants who regularly exercised (aOR: 0.48 [95% CI: 0.31-0.75]; *P* < 0.001), with high income (aOR: 0.31 [95% CI: 0.14-0.69]; *P* = 0.004) and with at least a college degree compared to participants with less than a college degree (aOR: 0.74 [95% CI: 0.64-0.87]; *P* < 0.001). We also examined the difference in the mean duration between the administration of questionnaires and the diagnosis of MI. The time since MI duration was found to be 14.5 years for individuals with MI and psychological distress, compared to 13.0 years for those with MI without psychological distress (*P* = 0.03).

## Discussion

In this study, we identified a 2.5-fold higher prevalence of psychological distress among adults with MI compared to those without MI. Notably, the prevalence fluctuated over time, with a surge in 2021, potentially linked to the COVID-19 pandemic. This prevalence of psychological distress was higher in younger individuals, females, Hispanic persons, and those with lower socioeconomic status. Psychological distress in patients with MI was associated with a 2-fold increase in medical expenditures and health care utilization, with key predictors of psychological distress including sex, race, sociodemographic factors, and comorbidities. Protective factors included regular exercise and higher education, particularly college-level education (Central Illustration).

**Central Illustration:**
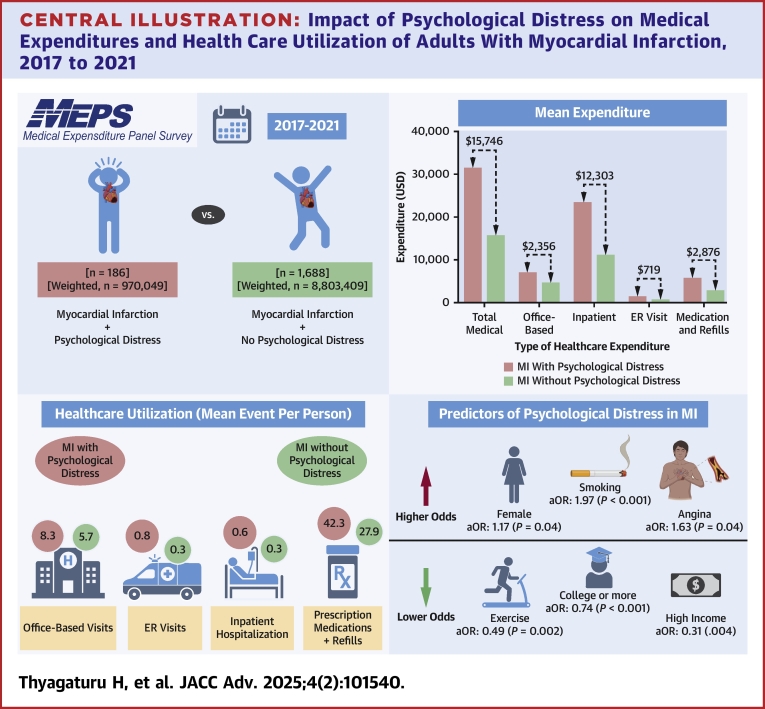
**Impact of Psychological Distress on Medical Expenditures and Health Care Utilization of Adults With Myocardial Infarction, 2017 to 2021** Summary of increased medical expenditures and health care utilization in myocardial infarction patients with psychological distress. Those with psychological distress show higher office visits, emergency room visits, inpatient stays, and prescription refills, as well as greater costs across total medical, inpatient, emergency room visit, and medication expenses. Predictors of psychological distress include female sex, smoking, and lower income, underscoring the added health care burden in this population. aOR = adjusted OR; ER = emergency room; other abbreviation as in Figure 1.

Our observed 9.3% prevalence of psychological distress among those with MI aligns with previous research, which shows a higher prevalence of psychiatric disorders in this population.^2^^,^^3^^,^^5^ The mechanisms contributing to this trend are multifactorial, potentially including dysregulation of the hypothalamic-pituitary-adrenal axis and inflammation,^3^ while also indirectly affecting psychological well-being due to the chronic nature of the disease.^19^^,^^20^ Demographic factors, such as higher prevalence of psychological distress in women and Hispanic individuals, are likely influenced by socioeconomic challenges, discrimination, and lifestyle factors.^21^, ^22^, ^23^ Additionally, financial burdens associated with MI, especially among those of low socioeconomic status, exacerbate distress.^24^^,^^25^ This finding is consistent with our observations, where we noted an association between psychological distress and patients who were uninsured or covered by public insurance. Furthermore, our analysis highlighted a significant increase in the prevalence of psychological distress among MI population in 2021, potentially related to the lockdowns and stressors associated with the COVID-19 pandemic, in addition to awareness of increased risk given established CAD and cardiovascular disease risk factors.^26^ This suggests an interplay between external stressors, public health policies, and mental health.^26^

We noted a protective association between higher education levels and reduced psychological distress, possibly linked to greater health literacy, improved financial stability, better insurance coverage, enhanced access to guideline-directed medical therapies, and greater medication adherence among college-educated individuals.^27^^,^^28^ In addition, our findings suggest that persons with a history of MI experiencing psychological distress tend to have irregular exercise patterns, raising the possibility of regular exercise serving as a protective factor in this group.^29^^,^^30^ However, it is essential to acknowledge the nuanced interpretation of these results, as this observation could alternatively indicate the severity of illness, potentially limiting regular exercise. This dual interpretation underscores the intricate interplay among physical activity, chronic illness, and psychological well-being.

The relationship between psychological distress and higher health care-related costs is observed across various diseases.^31^^,^^32^ After adjusting for chronic conditions, health behaviors, and sociodemographic factors, we observed higher rates of office-based visits, ER visits, hospital admissions, and prescribed medication use in adults with MI and psychological distress. This increase in health care utilization may stem from symptoms of psychological distress, such as anxiety and panic attacks, may be misinterpreted as ischemic symptoms, leading to excess health care costs and utilization.^33^^,^^34^ Additionally, psychological disorders are associated with endothelial dysfunction and heightened platelet reactivity,^8^^,^^9^^,^^35^ which exacerbates thrombosis risk, leading to a recurrence of myocardial ischemia and MI events.^35^ Moreover, the coexistence of cardiovascular disease and mental illness may contribute to disparities in assessment and treatment of anginal symptoms.^36^, ^37^, ^38^ This may lead to complications, recurrent MIs, and, consequently, poorer outcomes, triggering recurrent use of health care resources and increased expenditures down the line. Our findings highlight the need for integrated care approach that concurrently addresses the physical symptoms and psychological aspects of MI. By identifying the linkage between psychological distress, socioeconomic factors, and health-risk behaviors, such as reduced physical activity, our study points to critical areas for intervention. The implementation of a holistic population care strategy that encompasses behavioral, social, and economic considerations could positively influence affected population outcomes and reduce the overall health care costs associated with MI.

Our study showed that females had a higher likelihood of psychological distress compared to males. The finding is consistent with a previous NHIS study from 2009 to 2013 highlighting the increased susceptibility of women to serious psychological distress compared to men in all age groups,^39^ the etiology of which remains complex, influenced by a combination of social, cultural, and biological factors.^40^ Our study also shows that Black persons with a history of MI are less likely to be associated with psychological distress than White persons with MI. Watkins et al also highlighted a similar mental health race paradox in an NHIS-based cross-sectional study that Black persons had lower psychological distress despite the lower family income status.^41^ Black individuals, particularly those from historically marginalized communities, may be less likely to report symptoms of psychological distress due to stigma, mistrust of the health care system, and cultural barriers to disclosing mental health concerns.^42^ Cultural norms regarding emotional expression, coping styles, and help-seeking behaviors vary across racial and ethnic groups, influence the likelihood of acknowledging and disclosing mental health symptoms.^43^ Efforts to promote gender and racial/ethnic equity policies and enhance access to mental health services are essential to mitigate the impact of these disparities on mental health outcomes. Results from post hoc analysis showed no significant differences between time since MI and total expenditures in patients with or without psychological distress, suggesting that other factors, such as sociodemographic characteristics and health behaviors, may play a larger role in health care costs (Supplemental Tables 4 and 5).

### Study limitations

Our study provides valuable insights but has several limitations. First, the cross-sectional design limits our ability to establish causal relationships between psychological distress and its effects on medical expenditures and health care utilization. It is unclear if psychological distress precedes or results from increased health care demands, with reverse causality being a possibility. Second, while we examined total medication use, we did not assess specific medication classes, limiting our understanding of their influence on health care costs. Third, the data set lacked details on MI severity, treatments, and disease stages, which could affect psychological distress and health care utilization. Fourth, the Kessler questionnaire may not fully capture the complexity of psychological distress in patients with MI, given potential overlaps with somatic MI symptoms. Lastly, our study focused on health care costs without exploring qualitative patient experiences, which could offer deeper insights. Future research should adopt longitudinal designs to better understand the relationship between psychological distress, MI, and health care outcomes, using more comprehensive data for finer analysis.

## Conclusions

In summary, psychological distress persists among patients with MI long after their initial events, correlating with substantial health care utilization and medical expenses. Our study provides valuable insights into the specific need for targeted interventions in psychological care for the MI population. Early identification and treatment of psychological distress in patients with MI could lead to more favorable health outcomes, reducing morbidity and the overall burden of CAD. Implementing routine screenings to identify the requirement for psychological care can enable early intervention, ultimately lowering overall costs and health care utilization and contributing to enhanced outcomes for individuals with MI.

Perspectives **COMPETENCY IN PRACTICE-BASED LEARNING AND IMPROVEMENT:** This study highlights the significant association between psychological distress and increased health care utilization in patients with MI. Our findings emphasize the need for routine mental health screenings in this population to improve clinical outcomes and reduce health care costs.

**TRANSLATIONAL OUTLOOK:** Future research should focus on validating these findings in larger cohorts and exploring interventions that address psychological distress in patients with MI, potentially reducing the burden on health care systems.

## Funding support and author disclosures

The authors have reported that they have no relationships relevant to the contents of this paper to disclose.

## Footnotes

Appendix For the Kessler questionnaire, STROBE checklist, and supplemental tables, please see the online version of this paper.
