# Supplementary material for: Psychological Distress in Adults With Myocardial Infarction: Implications for Health Care Utilization and Expenditure
Source: JACC Adv. 2025 Jan 9;4(2):101540. doi: 10.1016/j.jacadv.2024.101540 (PMC11780088; doi:10.1016/j.jacadv.2024.101540)
Supplement: Supplementary appendix [file mmc1.docx]

**SUPPLEMENTAL APPENDIX**

**Kessler questionnaire**: Adopted from MEPS documentation file. Scores are recorded as 0, 1, 2 ,3, 4 based on the responses for the questionnaire as noted below. Psychiatric distress was defied as any score ≥13.


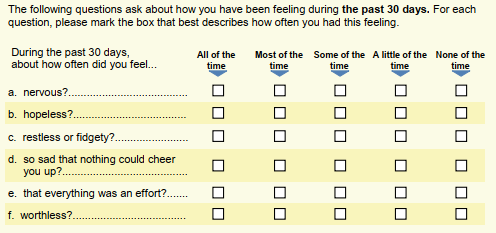


Average Kessler (K6) score for psychological distress in the MI population was 4.25, whereas it was 2.74 in the population without MI.

**Supplemental Table 1**: ICD-10-CM codes used in this study

| **Diagnosis** | **ICD-10-CM codes** |
| --- | --- |
| History of myocardial infarction | I21 and Priority condition* |
| Heart failure | I50 |
| Hypertension | I10 and priority condition* |
| Diabetes mellitus | Priority condition* |
| Chronic obstructive pulmonary disease | Priority condition* |
| High cholesterol | Priority condition* |
| Cancer | Priority condition* |
| Asthma | Priority condition* |
| Stroke | I63 and Priority condition* |
| Peripheral vascular disease | I73 |
| Sleep disorders | G47 |
| Obesity | E66 and BMI >25 kg/m^2^ |
| Depression | F32 |
| Anxiety | F41 |

*Priority conditions available in the MEPS dataset. During the survey, participants were asked yes or no questions about these conditions and were coded in the dataset as unique variables.

**Supplemental Table 2**: Prevalence of Psychological Distress Among Adults with and without Myocardial Infarction

| Year | Prevalence of psychological distress in MI, % (95% CI) | Prevalence of psychological distress in non-MI, % (95% CI) | p-value |
| --- | --- | --- | --- |
| 2017 | 11.77 (9.8 to 14) | 3.35 (3.0 to 3.5) |  |
| 2018 | 9.39 (7.6 to 11.2) | 3.35 (3.0 to 3.5) |  |
| 2019 | 7.87 (6.3 to 9.7) | 3.29 (2.9 to 3.5) |  |
| 2020 | 3.72 (2.1 to 5.5) | 3.92 (3.5 to 4.1) |  |
| 2021 | 10.55 (8.8 to 12.9) | 4.22 (3.9 to 4.4) |  |
| Total | 9.28 (7.8 to 11) | 3.6 (3.4 to 3.8) | < 0.001 |

MI = myocardial infarction

**
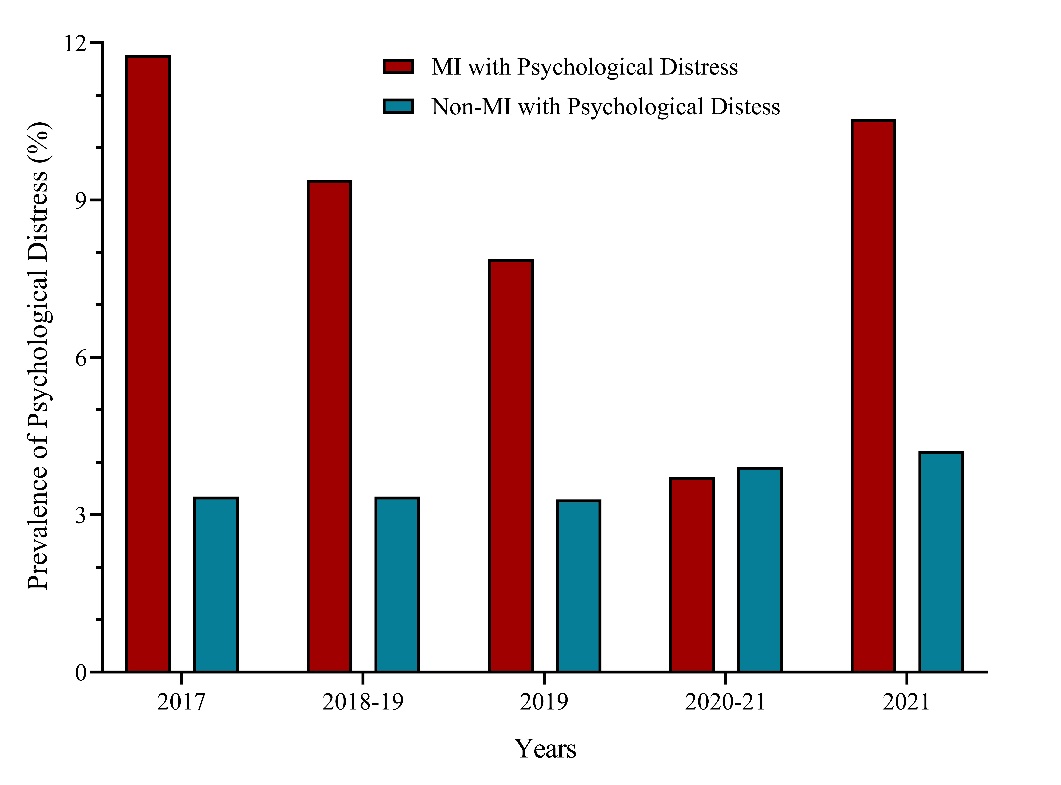
**

**Supplemental Table 3**: Association of Psychological Distress among Adults with Myocardial Infarction using Survey Estimates.

|  | Adjusted Odds of psychological distress, aOR (95% CI) | p-value |
| --- | --- | --- |
| **Age groups** |  |  |
| ≥85 | Ref. |  |
| 18-39 | 2.02 (0.57 to 7.14) | 0.27 |
| 40-64 | 2.29 (0.93 to 5.66) | 0.07 |
| 65-84 | 1.43 (0.59 to 3.47) | 0.42 |
| **Access to health care** |  |  |
| Private insurance | Ref. |  |
| Public | 1.71 (1.03 to 2.84) | .04 |
| Uninsured | 1.81 (0.60 to 5.43) | .29 |
| Having usual source of care | 1.35 (0.65 to 2.82) | .42 |
| **Health factors** |  |  |
| Exercise regularly | 0.49 (0.31 to 0.77) | .002 |
| Elevated BMI>25 kg/m2 | 1.81 (0.91 to 3.61) | .09 |
| Smoking | 1.97 (1.65 to 2.34) | < 0.001 |
| **Socio-demographics** |  |  |
| Female vs. Male | 1.17 (1.01 to 1.37) | .04 |
| **Education** |  |  |
| Less than college | Ref. |  |
| College or more | 0.74 (0.64 to 0.87) | < 0.001 |
| **Marital status** |  |  |
| Married | Ref. |  |
| Widowed | 1.25 (0.62 to 2.49) | .53 |
| Divorced | 1.41 (1.15 to 1.73) | .001 |
| Never married | 0.60 (0.30 to 1.21) | .15 |
| **Race/ethnicity** |  |  |
| White | Ref. |  |
| Black | 0.54 (0.44 to 0.66) | 0.001 |
| Hispanic | 0.84 (0.67 to 1.05) | .14 |
| Asian and other | 1.09 (0.76 to 1.57) | .61 |
| **Income** |  |  |
| Poor | Ref. |  |
| Near poor | 0.65 (0.35 to 1.22) | .18 |
| Low income | 0.46 (0.27 to 0.80) | .006 |
| Middle income | 0.38 (0.22 to 0.67) | .001 |
| High income | 0.31 (0.14 to 0.69) | .004 |
| **Comorbidities** |  |  |
| Coronary heart disease | 1.13 (0.70 to 1.83) | .62 |
| Angina | 1.63 (1.03 to 2.59) | .04 |
| Hypertension | 1.06 (0.59 to 1.93) | .84 |
| Diabetes mellitus | 0.78 (0.52 to 1.17) | .23 |
| COPD | 0.86 (0.49 to 1.53) | .62 |
| High cholesterol | 1.28 (0.75 to 2.17) | .36 |
| Cancer | 0.92 (0.57 to 1.49) | .73 |
| Asthma | 1.53 (0.92 to 2.55) | .10 |

OR = odds ratio; COPD = chronic obstructive pulmonary disease; BMI = body mass index

**Supplemental Table 4:** Outcome psychological distress and exposure Time since MI adjusted for sociodemographic factors and using survey metrics (logistic regression)

| Psychological distress | Odds ratio | P value | Lower CI | Upper CI |
| --- | --- | --- | --- | --- |
| Time since MI | .9856617 | 0.12 | .96 | 1.00 |

MI = myocardial infarction

**Interpretation:** Time since diagnosis was not significantly associated with psychological distress in MI (p=0.121)

**Supplemental Table 5:** Outcome expenditures and exposures time since MI and psychological distress and their interaction adjusted for sociodemographic factors and using survey metrics (gamma regression).

| Total Expenditure | Coefficient | Standard Error | P value | Lower CI | Upper CI |
| --- | --- | --- | --- | --- | --- |
| Time since MI | -0.003 | 0.004 | 0.367 | -0.01 | 0.004 |
| Psychological distress | 0.73 | 0.22 | 0.001 | 0.29 | 1.18 |
| Interaction between psychological distress and MI | -0.007 | 0.02 | 0.750 | -0.05 | 0.03 |

MI = myocardial infarction

**Interpretation:** Total expenditures were not significantly associated with time since diagnosis of MI (p=0.367). There was no interaction effect between time since diagnosis and psychological distress on expenses among MI sample (p=0.750).

**STROBE Checklist**

|  | Item No | Recommendation | Page No |
| --- | --- | --- | --- |
| **Title and abstract** | 1 | 1. Indicate the study’s design with a commonly used term in the title or the abstract   *We conducted a cross-sectional observational study of the 2017-2021 Medical Expenditure Panel Survey (MEPS) to identify adults with history of MI.* | 2 |
|  |  | 1. Provide in the abstract an informative and balanced summary of what was done and what was found   *Abstract in this study consists of background, method, results and conclusion sections with informative and balanced information.* | 2-3 |
| Introduction | | | |
| Background/rationale | 2 | Explain the scientific background and rationale for the investigation being reported  *Myocardial infarction (MI) is common and costly. Psychological distress in MI patients can worsen outcomes and increase healthcare costs, yet its impact on healthcare utilization and expenditure is underexplored. This study investigates these associations to inform potential interventions.* | 4 |
| Objectives | 3 | State specific objectives, including any prespecified hypotheses  *To examine the association between psychological distress and healthcare utilization and expenditure in adults with a history of myocardial infarction.* | 4 |
| Methods | | | |
| Study design | 4 | Present key elements of study design early in the paper  *This is a cross-sectional observational study using data from the 2017-2021 Medical Expenditure Panel Survey (MEPS). The study identifies adults with a history of myocardial infarction (MI) and stratifies them by the presence or absence of psychological distress* | 5-6 |
| Setting | 5 | Describe the setting, locations, and relevant dates, including periods of recruitment, exposure, follow-up, and data collection  *The study utilizes data from the Medical Expenditure Panel Survey (MEPS), a nationally representative survey conducted in the United States. Data were collected from January 2017 to December 2021. MEPS includes in-person and virtual interviews with non-institutionalized U.S. residents, capturing healthcare usage, expenditures, demographics, and health conditions.* | 5-6 |
| Participants | 6 | (*a*) Give the eligibility criteria, and the sources and methods of selection of participants  *Adults aged 18 years or older with a history of myocardial infarction (MI) were identified using MEPS survey responses and the ICD-10 code "I21." The study excluded participants with missing data on psychological distress. MEPS employs a nationally representative sampling framework of non-institutionalized U.S. residents. As a retrospective analysis of survey data, there was no active follow-up, and participant data were collected through five interview rounds over two years per MEPS panel, allowing assessment of healthcare utilization and expenditures.* | 5-6 |
| Variables | 7 | Clearly define all outcomes, exposures, predictors, potential confounders, and effect modifiers. Give diagnostic criteria, if applicable  *The primary outcomes are healthcare expenditures (total and by setting) and healthcare utilization (number of visits/services used). The exposure is psychological distress, measured by the Kessler (K6) questionnaire, with scores ≥13 indicating distress. Predictors include demographic (age, sex, race, education, income) and health-related factors (insurance, marital status, comorbidities). Key confounders considered are age, sex, income, insurance, and comorbidities, while effect modifiers like sex, race, and socioeconomic status were evaluated for their influence on distress and healthcare outcomes. Diagnostic criteria for distress used the K6 score (≥13), and myocardial infarction was identified by ICD-10 code "I21."* | 6-7 |
| Data sources/ measurement | 8* | For each variable of interest, give sources of data and details of methods of assessment (measurement). Describe comparability of assessment methods if there is more than one group  *Each variable of interest was derived from the Medical Expenditure Panel Survey (MEPS) dataset, which provides standardized data for healthcare utilization, expenditures, and demographic factors. MEPS standardizes data collection across all participants, using the same assessment tools and definitions, ensuring comparability between groups with and without psychological distress.* | *5-7* |
| Bias | 9 | Describe any efforts to address potential sources of bias  *To address potential sources of bias, the study used survey weights provided by MEPS to produce nationally representative estimates, minimizing selection bias. Participants with missing data on psychological distress were excluded to ensure data completeness. Additionally, multivariable regression models were used to adjust for potential confounding variables, such as age, sex, income, and comorbidities, which could influence both psychological distress and healthcare utilization. These steps helped reduce the influence of systematic differences between groups on the study outcomes.* | 5-7 |
| Study size | 10 | Explain how the study size was arrived at  *The study size was determined based on available data from the 2017-2021 Medical Expenditure Panel Survey (MEPS). Initially, all adults with a history of myocardial infarction (MI) were identified. After excluding individuals with missing data on psychological distress, the final sample included 44,716 participants. This sample size reflects the MEPS dataset's nationally representative structure, enabling reliable estimation of associations between psychological distress and healthcare utilization and expenditures in the MI population.* | 5-6 |
| Quantitative variables | 11 | Explain how quantitative variables were handled in the analyses. If applicable, describe which groupings were chosen and why  *Quantitative variables in the study, such as healthcare expenditures and utilization metrics (e.g., total medical costs, number of office visits, ER visits), were analyzed as continuous variables to capture precise differences between groups. For demographic factors like income, categorical groupings were used (e.g., Federal Poverty Level categories) to account for socioeconomic disparities. Age was also grouped to assess variations across life stages, facilitating clearer comparisons between younger and older adults. These groupings were chosen to enhance interpretability and relevance in examining the association between psychological distress and healthcare outcomes.* | 7-8 |
| Statistical methods | 12 | (*a*) Describe all statistical methods, including those used to control for confounding  *The study employed multivariable regression models to assess the relationship between psychological distress and healthcare utilization and expenditures, with adjustments for confounding variables. For continuous outcomes, such as healthcare expenditures, generalized linear models with a gamma distribution and log link were applied to accommodate skewed data. For count outcomes, such as the number of healthcare visits, negative binomial regression was used to account for overdispersion. Zero-inflated negative binomial models were employed for rare events, such as inpatient and ER visits, to capture both the high frequency of non-events and the counts among those who used these services. To control for confounding, each model adjusted for variables likely to influence both psychological distress and healthcare outcomes, including age, sex, race, income, education, insurance status, and comorbidities. Survey weights from MEPS were incorporated to account for the survey’s complex sampling design, ensuring that findings were nationally representative.* | 7-9 |
|  |  | (*b*) Describe any methods used to examine subgroups and interactions  *The study examined subgroups by stratifying participants based on key demographic variables, including age, sex, race, and socioeconomic status, to assess variations in the association between psychological distress and healthcare outcomes across these groups. Additionally, interaction terms were incorporated in the regression models to evaluate whether the relationship between psychological distress and healthcare utilization or expenditures differed significantly by demographic characteristics. This approach allowed for identifying any differential effects of psychological distress on healthcare outcomes within specific population segments.* | 7-9 |
|  |  | (*c*) Explain how missing data were addressed  *Missing data were handled by excluding participants with incomplete information.* | 9 |
|  |  | (*d*) If applicable, describe analytical methods taking account of sampling strategy  *Given the cross-sectional design and complex sampling strategy of the MEPS dataset, survey weights, clustering, and stratification provided by MEPS were applied in all analyses to produce nationally representative estimates.* | 9 |
|  |  | (*e*) Describe any sensitivity analyses  *No sensitivity analysis done.* |  |
| Results | | | |
| Participants | 13* | (a) Report numbers of individuals at each stage of study—eg numbers  *potentially eligible, examined for eligibility, confirmed eligible, included in the study, completing follow-up, and analyzed*  *The study began with an initial dataset of 79,871 observations from the 2017-2021 MEPS which were examined for eligibility. 1,874 were confirmed eligible, included in the study and analyzed.* | 9-10 |
|  |  | (b) Give reasons for non-participation at each stage  *Non-participation occurred at two main stages: first, 19,651 observations were excluded due to inapplicability or non-response to the myocardial infarction (MI) diagnosis question, leaving 60,220 participants with MI data. Second, among these, 15,504 observations were excluded due to inapplicability or non-response to the psychological distress assessment (Kessler K6 questionnaire). These exclusions ensured that only participants with complete data on both MI status and psychological distress were included in the final analysis.* | 9 |
|  |  | (c) Consider use of a flow diagram  *Figure 1* | 9 |
| Descriptive data | 14* | (a) Give characteristics of study participants (eg demographic, clinical, social) and information on exposures and potential confounders  *Characteristics of study participants Given in Table 1* | 9-10 |
|  |  | (b) Indicate number of participants with missing data for each variable of interest  *The total proportion of missing values were <3%.* | 9-10 |
| Outcome data | 15* | Report numbers of outcome events or summary measures  Outcome events reported in the results sections. | 10-11 |
| Main results | 16 | (*a*) Give unadjusted estimates and, if applicable, confounder-adjusted estimates and their precision (eg, 95% confidence interval). Make clear which confounders were adjusted for and why they were included  *The study reported confounder-adjusted estimates for the association between psychological distress and healthcare utilization and costs. Adjusted estimates controlled for confounders such as age, sex, race, income, education, insurance, and comorbidities, with results remaining significant. 95% confidence intervals were provided.* | 10-11 |
|  |  | (*b*) Report category boundaries when continuous variables were categorized  *In this study, continuous variables were categorized to improve the interpretability of results. Age was divided into groups representing life stages (18-34, 35-54, 55-74, and 75+ years) to facilitate age-related comparisons. Income was categorized according to Federal Poverty Level (FPL) thresholds, defining groups as poor/negative (≤100% FPL), near poor (100%-125% FPL), low income (125%-200% FPL), middle income (200%-400% FPL), and high income (>400% FPL). Additionally, Body Mass Index (BMI) was grouped using clinical cutoffs to distinguish normal weight, overweight, and obese participants. These categorizations allowed clearer insights into the relationships between demographic factors, psychological distress, and healthcare utilization and expenditures (Table 1)* | 9-10 |
|  |  | (*c*) If relevant, consider translating estimates of relative risk into absolute risk for a meaningful time period  *Not Relevant* |  |
| Other analyses | 17 | Report other analyses done—eg analyses of subgroups and interactions, and sensitivity analyses  *logistic regression analysis was conducted to identify factors associated with psychological distress among patients with a history of myocardial infarction (MI). The analysis included a range of demographic, socioeconomic, and clinical variables to examine their relationships with psychological distress.* | 11 |
| Discussion | | | |
| Key results | 18 | Summarize key results with reference to study objectives  *Key results summarized in the first paragraph of the discussion section.* | 11 |
| Limitations | 19 | Discuss limitations of the study, taking into account sources of potential bias or imprecision. Discuss both direction and magnitude of any potential bias  *Discussed in the limitation section of the discussion.* | 14-15 |
| Interpretation | 20 | Give a cautious overall interpretation of results considering objectives, limitations, multiplicity of analyses, results from similar studies, and other relevant evidence  *The discussion provides a cautious interpretation of results, aligning with study objectives and recognizing limitations like the cross-sectional design, which restricts causal inferences, and the absence of data on MI severity and treatment, which may impact results. It notes that the Kessler (K6) tool may underestimate distress effects and highlights that consistent findings across subgroup and sensitivity analyses strengthen the results. Comparisons with similar studies support the association between psychological distress and increased healthcare use, suggesting the value of integrating mental health care in MI management.* | 14 |
| Generalizability | 21 | Discuss the generalizability (external validity) of the study results  *The sample comes from the MEPS dataset, which is designed to be representative of the non-institutionalized U.S. population.* | 6 |
| Other information | | | |
| Funding | 22 | Give the source of funding and the role of the funders for the present study and, if applicable, for the original study on which the present article is based  No funding received for this study (Title Page). | 1 |

*Give information separately for exposed and unexposed groups.
